# Applying Compressed Sensing Volumetric Interpolated Breath-Hold Examination and Spiral Ultrashort Echo Time Sequences for Lung Nodule Detection in MRI

**DOI:** 10.3390/diagnostics12010093

**Published:** 2021-12-31

**Authors:** Yu-Sen Huang, Emi Niisato, Mao-Yuan Marine Su, Thomas Benkert, Ning Chien, Pin-Yi Chiang, Wen-Jeng Lee, Jin-Shing Chen, Yeun-Chung Chang

**Affiliations:** 1 Department of Medical Imaging, National Taiwan University Hospital and National Taiwan University College of Medicine, Taipei 100, Taiwan; yusenh@ntu.edu.tw (Y.-S.H.); 007219@ntuh.gov.tw (M.-Y.M.S.); 104750@ntuh.gov.tw (N.C.); 113054@ntuh.gov.tw (P.-Y.C.); 004582@ntuh.gov.tw (W.-J.L.); 2 Department of Radiology, National Taiwan University College of Medicine, Taipei 100, Taiwan; 3 Siemens Healthcare Limited, Taipei 100, Taiwan; emi.niisato@gmail.com; 4 Siemens Healthcare GmbH, 91052 Erlangen, Germany; benkert.thomas@siemens-healthineers.com; 5 Department of Surgery, National Taiwan University Hospital and National Taiwan University College of Medicine, Taipei 100, Taiwan; chenjs@ntu.edu.tw

**Keywords:** spiral UTE, compressed sensing, VIBE, lung nodule

## Abstract

This prospective study aimed to investigate the ability of spiral ultrashort echo time (UTE) and compressed sensing volumetric interpolated breath-hold examination (CS-VIBE) sequences in magnetic resonance imaging (MRI) compared to conventional VIBE and chest computed tomography (CT) in terms of image quality and small nodule detection. Patients with small lung nodules scheduled for video-assisted thoracoscopic surgery (VATS) for lung wedge resection were prospectively enrolled. Each patient underwent non-contrast chest CT and non-contrast MRI on the same day prior to thoracic surgery. The chest CT was performed to obtain a standard reference for nodule size, location, and morphology. The chest MRI included breath-hold conventional VIBE and CS-VIBE with scanning durations of 11 and 13 s, respectively, and free-breathing spiral UTE for 3.5–5 min. The signal-to-noise ratio (SNR), contrast-to-noise ratio (CNR), and normal structure visualizations were measured to evaluate MRI quality. Nodule detection sensitivity was evaluated on a lobe-by-lobe basis. Inter-reader and inter-modality reliability analyses were performed using the Cohen κ statistic and the nodule size comparison was performed using Bland–Altman plots. Among 96 pulmonary nodules requiring surgery, the average nodule diameter was 7.7 ± 3.9 mm (range: 4–20 mm); of the 73 resected nodules, most were invasive cancer (74%) or pre-invasive carcinoma in situ (15%). Both spiral UTE and CS-VIBE images achieved significantly higher overall image quality scores, SNRs, and CNRs than conventional VIBE. Spiral UTE (81%) and CS-VIBE (83%) achieved a higher lung nodule detection rate than conventional VIBE (53%). Specifically, the nodule detection rate for spiral UTE and CS-VIBE reached 95% and 100% for nodules >8 and >10 mm, respectively. A 90% detection rate was achieved for nodules of all sizes with a part-solid or solid morphology. Spiral UTE and CS-VIBE under-estimated the nodule size by 0.2 ± 1.4 mm with 95% limits of agreement from −2.6 to 2.9 mm and by 0.2 ± 1.7 mm with 95% limits of agreement from −3.3 to 3.5 mm, respectively, compared to the reference CT. In conclusion, chest CT remains the gold standard for lung nodule detection due to its high image resolutions. Both spiral UTE and CS-VIBE MRI could detect small lung nodules requiring surgery and could be considered a potential alternative to chest CT; however, their clinical application requires further investigation.

## 1. Introduction

Computed tomography (CT) is currently the gold standard for high-resolution imaging of the lung for detection of lung nodules and assessment of pulmonary disease [1,2]. Due to its rapid scanning time and high image quality, chest CT has been incorporated in clinical settings for the diagnosis of lung diseases and early detection of lung malignancies. However, radiation dose exposure remains a disadvantage of chest CT [3,4,5]. Magnetic resonance imaging (MRI) provides a radiation-free modality for lung imaging, and advancements in MRI techniques have enabled the visualization of lung tissues [6,7]. Among the various MRI techniques, volumetric interpolated breath-hold examination (VIBE) has achieved favorable ratings for the detection of pulmonary nodules under 1.5 T and 3 T conditions. Non-contrast controlled aliasing in parallel imaging results in higher acceleration VIBE, which could detect small lung nodules with acceptable image quality, facilitating a short breath-holding time for three-dimensional image acquisition [8].

Compressed sensing (CS) VIBE is an important sequence that has recently been applied to advance MRI scans. CS-VIBE allows image acquisition with high spatial and temporal resolution during a short breath-hold scan time. Now integrated into routine clinical practice for abdomen, breast, brain, and cardiac MRI [9,10,11], its application to lung images is currently under investigation. In lung imaging, CS-VIBE has been used for dynamic MRI, pulmonary ventilation, and lung tumor movement evaluation [12,13]. The detection of small lung nodules by CS-VIBE has yet to be studied. 

Spiral ultrashort echo time (UTE) sequence, equipped with short echo time (TE, 0.05 ms) advancement, has now been developed to detect small lung nodules with the advantage of the short image acquisition time, thereby eliminating needless breath-holding during scanning [14,15,16]. Nevertheless, the spiral UTE sequence is still under development, is selectively equipped in existing machines, and is not accessible in all circumstances. 

Several studies have been conducted regarding the use of lung MRI sequences in the detection of small lung nodules, and the detection rate was shown to be above 80% for novel MRI sequences [15,16,17]. To the best of our knowledge, no study has simultaneously evaluated the capability of conventional VIBE, CS-VIBE, or spiral UTE sequences in lung imaging. Therefore, this study aimed to compare the image quality and small nodule detection of breath-hold CS-VIBE and free-breathing spiral UTE sequences with the conventional breath-hold VIBE sequence and the gold standard chest CT images.

The novelties of this work are presented below:

- The pulmonary nodules detected by the imaging modalities, including CT and MRI, were scheduled for VATS resection, and pathology correlation (malignant vs. non-malignant) of the nodules was performed.
- Both UTE and CS-VIBE sequences provide radiation-free pulmonary nodule detection, which is suitable for young people, pregnant women, patients requiring serial and longitudinal follow-up, or people unwilling to undergo radiation exposure.
- Variable respiratory motion management (breath-hold for CS-VIBE with scanning durations of 13 s vs. free-breathing for spiral UTE with scanning durations of 3.5–5 min) was investigated.

## 2. Materials and Methods

### 2.1. Study Design

**Figure 1:**
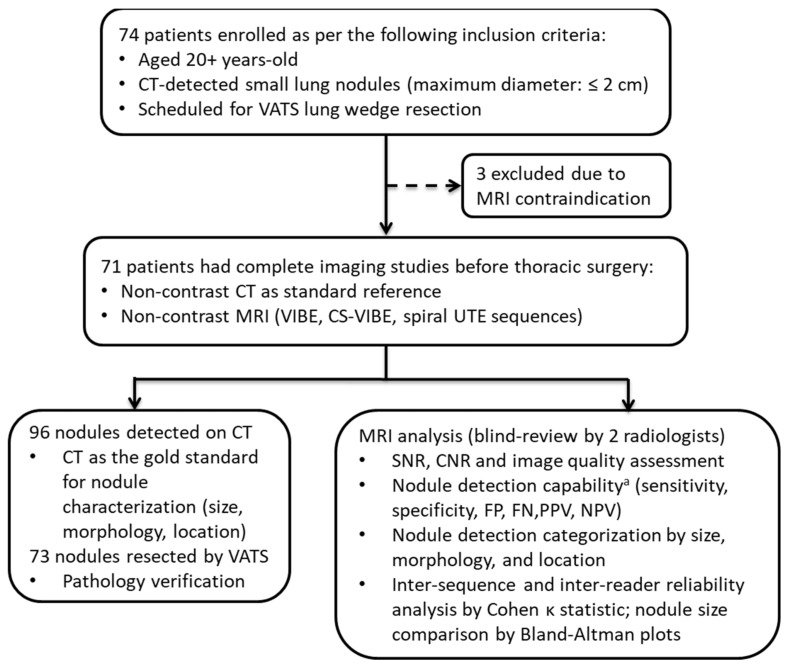
Flowchart of the study. ^a^ Radiologists recorded the presence or absence of nodules on a lobe-by-lobe basis. Abbreviations: CT: computed tomography; VATS: video-assisted thoracoscopic surgery; MRI: magnetic resonance imaging; CS: compressed sensing; VIBE: volumetric interpolated breath-hold examination; UTE: ultrashort echo time; SNR: signal-to-noise ratio; CNR: contrast-to-noise ratio; FP: false positive; FN: false negative; PPV: positive predictive value; NPV: negative predictive value.

Between January 2019 and December 2019, 74 patients above 20 years old, with small lung nodules detectable on CT imaging (maximum diameter: ≤2 cm), scheduled for video-assisted thoracoscopic surgery (VATS) lung wedge resection, were prospectively enrolled [18,19]. Three patients with MRI contraindication (metallic implants, claustrophobia, MRI-incompatible pacemakers, or MRI-incompatible prosthetic heart valves) who could not undergo MR examination were excluded from the study. A total of 71 patients underwent non-contrast chest CT and non-contrast MRI on the same day of or the day preceding thoracic surgery. Chest MRIs included conventional VIBE and CS-VIBE sequences, both with breath-holding procedures, and free-breathing spiral UTE sequences. CT was performed as the reference standard. A flow chart is shown in Figure 1.

### 2.2. MRI Acquisition

All lung MR images were obtained before VATS lung wedge resection using a 1.5 T scanner (MAGNETOM Aera, Siemens Healthcare, Erlangen, Germany). A 30-channel body matrix coil was placed directly on the thorax of each patient. The parameters of the conventional VIBE, spiral UTE, and CS-VIBE sequences are listed in Table 1. All patients underwent scanning in the headfirst supine position. The MRI protocols were validated in previous studies by our team and others [15,17,20,21].

### 2.3. CT Examinations

Whole lung CT without intravenous contrast was performed with a 128-slice CT scanner (Brilliance iCT, Philips Healthcare, Cleveland, OH, USA) with 120 kVp, 107–235 mA (modulated), collimation 0.625 mm, and images reconstructed at 1 mm thickness using filtered back projection (filter B). All patients were imaged whilst in the supine position while holding their breath at full inspiration. The average estimated radiation dose ranged from 2 to 7 mSv [2,5,22].

### 2.4. Image Analysis

Two radiologists (both specialized in cardiopulmonary imaging, with 23 years and 9 years of experience in evaluating chest MRI) were blinded to the exact location of the lung nodules. CT was considered the reference standard for determining the nodule size, location, and morphology (i.e., non-solid, part-solid, or solid), with the two radiologists in consensus. For the determination of nodule morphology, a non-solid nodule manifested as a hazy area of increased signal intensity in the lung that did not obliterate the bronchial or vascular margins, whereas part-solid nodules consisted of both ground-glass and solid soft tissue signal intensity components, and solid nodules had homogenous soft-tissue signal intensity [15,23]. 

CT and MR images were analyzed separately at 1-week intervals to avoid consecutive readings of the same patient, using the PACS system (IMPAX 6.0, AGFA Healthcare, Mortsel, Belgium). Each sequence was independently evaluated by the two radiologist readers, blinded both to the exact location of the lung nodules and the number of true positive lung nodules per patient. As all lung MR images were obtained from patients scheduled to undergo thoracic VATS surgery, the radiologist readers were aware that only patients with lung nodules would have undergone MRI scanning. The readers were asked to record the presence or absence of nodules on a lobe-by-lobe basis for each MR image dataset. When there were multiple nodules in one lobe, only one dominant nodule was included in the analysis.

The signal-to-noise ratio (SNR) and contrast-to-noise ratio (CNR) in selected regions of interest (ROIs) were calculated for the quantitative comparison of lung MRI quality between sequences [15,24]. The two radiologists independently drew circular ROIs in the target lung nodule, lung parenchyma, and trachea lumen on each sequence. ROI size was first adapted to the diameter of the target lung nodule, and the same ROI size was sequentially applied to the adjacent lung parenchyma and trachea lumen. Vascular markings and fissures were avoided when measuring the signal intensity (SI) of the lung parenchyma. The standard deviation of the SI measured in the tracheal lumen was considered as noise. SNR and CNR ratios were calculated using the following formulae: SNR = SI _nodule_/noise and CNR = [SI _nodule_ − SI _lung parenchyma_]/noise. 

For the qualitative assessment of normal structures, the two radiologists independently evaluated the images in each sequence. The criteria for visual assessment were followed as previously reported [14,15,25]. The score for each category was rated using a five-point scale regarding the visualization of normal structures, degree of noise and artifacts, and overall acceptability (Supplementary Table S1).

### 2.5. Statistical Analysis

Statistical analyses were performed using the Statistical Package for Social Sciences for Windows (version 17.0; SPSS, Chicago, IL, USA). For the quantitative assessment of SI and the qualitative assessment of normal structures, the average score of the two readers was presented, and a Student’s paired-samples *t*-test was used to evaluate the image quality scores of the sequences [15,24]. The sensitivity, specificity, false-positive rate, false-negative rate, positive predictive value, and negative predictive value were evaluated based on lobe-by-lobe analysis [16,21]. McNemar’s test was used to compare the sensitivity of the two sequences for nodule detection. Inter-sequence and inter-reader agreements were determined using the unadjusted Cohen κ statistic, with the following predefined levels of agreements: poor: ≤0.20, fair: 0.21–0.40, moderate: 0.41–0.60, substantial: 0.61–0.80, and almost perfect agreement: ≥0.81. Bland–Altman plots were generated to visually depict inter-reader and inter-modality variances and limits of agreement [16].

## 3. Results

### 3.1. Patient and Nodule Characteristics

The demographic data and nodule characteristics of the study population are presented in Table 2. The average diameter of the 96 nodules was 7.7 ± 3.9 mm (range: 4–20 mm). Of the 96 nodules, 73 (76%) were resected using VATS resection and then examined by a thoracic pathologist. The surgical pathology of the resected nodules was mostly invasive or pre-invasive cancer, as follows: invasive adenocarcinoma (37%), minimally invasive adenocarcinoma (36%), squamous cell carcinoma (1%), or adenocarcinoma in situ (15%).

### 3.2. Qualitative Assessment of Normal Structures and Nodules

As shown in Table 3, compared to the conventional VIBE sequence, spiral UTE and CS-VIBE images achieved higher subjective image quality scores for depicting pulmonary vascular structures and airways, with less image noise for nodule detection, leading to significantly higher overall image quality scores. Spiral UTE and CS-VIBE achieved equivalent scores for pulmonary vascular structures and airway depiction, image noise for nodule detection, and overall image quality.

Notably, CS-VIBE achieved non-significant lower scores (3.1 ± 0.6) for cardiac motion artifacts than spiral UTE (3.8 ± 0.4, *p* = 0.054), which did not differ from that of conventional VIBE (3.3 ± 0.6, *p* = 0.056). When a score ≥3 was considered acceptable for cardiac motion artifacts, 13% of the CS-VIBE cases showed notable cardiac motion artifacts, compared to 8% of the conventional VIBE and 3% of the spiral UTE cases.

### 3.3. Quantitative Assessment by SNR and CNR

**Figure 2:**
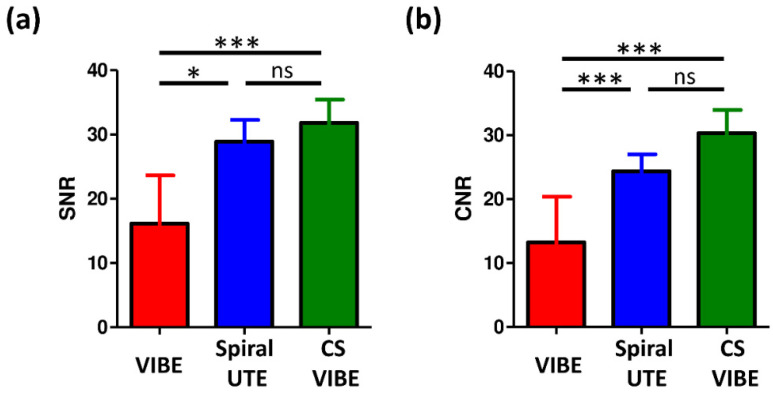
Comparison of (**a**) signal-to-noise ratio and (**b**) contrast-to-noise ratio in the quantitative assessment of nodule intensities between conventional VIBE, spiral UTE, or CS-VIBE sequences in lung MRI. Data are presented as means and standard deviations. Abbreviations: MRI: magnetic resonance imaging; CS: compressed sensing; VIBE: volumetric interpolated breath-hold examination; UTE: ultrashort echo time; SNR: signal-to-noise ratio; CNR: contrast-to-noise ratio; ns: not significant. * *p* < 0.05 and *** *p* < 0.001.

As shown in Figure 2a, the SNR of the nodules was significantly higher in both spiral UTE (*p* = 0.047) and CS-VIBE sequences (*p* < 0.001) than in conventional VIBE images, whereas no significant differences were noted between CS-VIBE and spiral UTE sequences (*p* = 0.398). After accounting for normal pulmonary parenchyma intensities, as shown in Figure 2b, the CNR of the nodules was significantly higher in both spiral UTE (*p* < 0.001) and CS-VIBE sequences (*p* < 0.001), compared to conventional VIBE images; comparable CNR values were observed between CS-VIBE and spiral UTE sequences (*p* = 0.161).

### 3.4. Evaluation of Nodule Detection Sensitivity

As shown in Table 4, the overall nodule detection sensitivity was significantly greater in spiral UTE and CS-VIBE compared with conventional VIBE (average values for spiral UTE: 81%, CS-VIBE: 83%, and VIBE: 53%, by two readers). The sensitivity of nodule detection did not differ significantly between spiral UTE and CS-VIBE.

**Figure 3:**
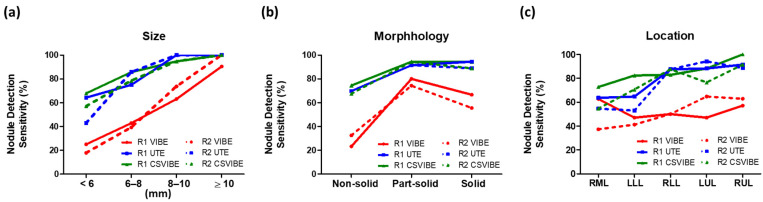
Nodule detection sensitivity for MRI categorized by (**a**) nodule diameter, (**b**) nodule morphology, and (**c**) location, as measured using CT. The nodule detection rate for spiral UTE and CS-VIBE sequences reached 95% when nodule size was >8 mm and reached 100% when nodule size was >10 mm; 90% detection was achieved when nodules possessed a part-solid or solid morphology for all sizes and improved in LLL and RML in both spiral UTE and CS-VIBE images. Abbreviations: MRI: magnetic resonance imaging; R1: reader 1; R2: reader 2; CS: compressed sensing; VIBE: volumetric interpolated breath-hold examination; UTE: ultrashort echo time; LML: left middle lobe; RML: right middle lobe.

**Figure 4:**
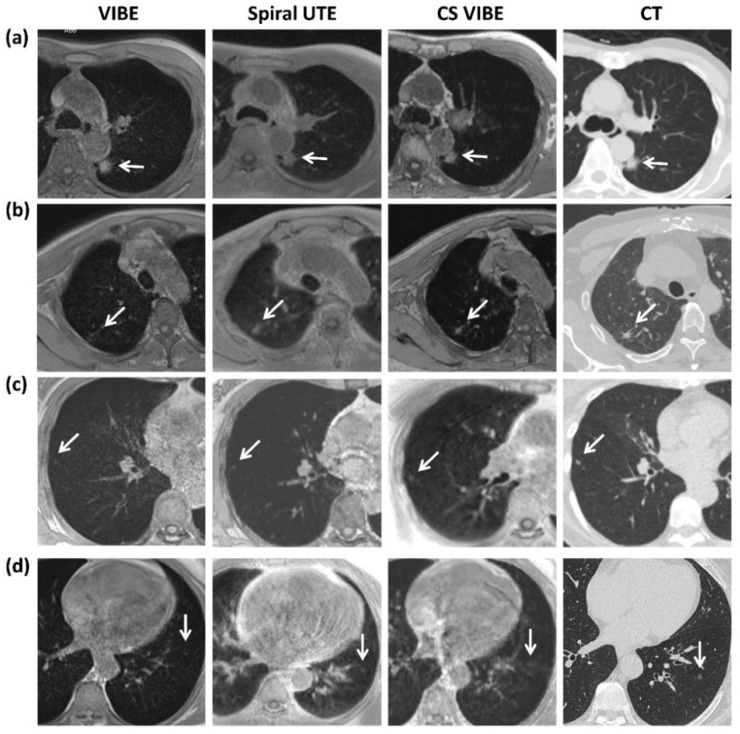
(**a**) Example of a 63-year-old man with an 18 mm part-solid nodule (white arrow) in the left lower lung shown on a reference axial CT image, detected clearly on conventional breath-hold VIBE, free-breathing spiral UTE MR, and breath-hold CS-VIBE images. (**b**) Example of a 66-year-old man with a 6 mm solid nodule (white arrow) in the right upper lung shown on a reference axial CT image, blurred on conventional breath-hold VIBE, and clearly depicted on free-breathing spiral UTE MR and breath-hold CS-VIBE images. (**c**) Example of a 55-year-old woman with a 4 mm solid nodule (white arrow) in the right lower lung shown on a reference axial CT image, detected vaguely on conventional breath-hold VIBE, and clearly depicted on free-breathing spiral UTE MR and breath-hold CS-VIBE images. (**d**) Example of a 60-year-old woman with a 4 mm non-solid nodule (white arrow) in the left lower lung shown on a reference axial CT image, detected on free-breathing spiral UTE, which could be easily missed on conventional breath-hold VIBE or CS-VIBE images due to cardiac pulsation artifacts. Abbreviations: MR: magnetic resonance; VIBE: volumetric interpolated breath-hold examination; UTE: ultrashort echo time; CS: compressed sensing.

As shown in Figure 3a, for both spiral UTE and CS-VIBE sequences, the nodule detection rate was low for pulmonary nodules <6 mm (average values for spiral UTE: 54% and CS-VIBE: 63%, by two readers). The nodule detection rate was creditable for nodule sizes of 6–8 mm (average values for spiral UTE: 80% and CS-VIBE: 82%, by two readers), reliable when the nodule size was 8–10 mm (average values for spiral UTE: 100% and CS-VIBE: 95%, by two readers), and reached 100% when the nodule size was ≥10 mm. Representative images are shown in Figure 4a–c.

As shown in Figure 3b, the nodule detection rate was yet to be improved in the non-solid morphology group (average values for spiral UTE: 70% and CS-VIBE: 71%, by two readers), and trustworthy in nodules with part-solid (average values for spiral UTE: 91% and CS-VIBE: 94%, by two readers) and solid morphology (average values for both spiral UTE and CS-VIBE: 92%, by two readers). 

In both spiral UTE and CS-VIBE images, a relatively unfavorable detection rate was observed in the right middle lobe (RML, average 64% for spiral UTE and 59% for CS-VIBE, by two readers) and left lower lobe (LLL, average 59% for spiral UTE and 77% for CS-VIBE, by two readers) (Figure 3c). The yet-to-be improved detection rate in the LLL and RML by CS-VIBE imaging may be partly attributed to pulmonary motions and the adjoining cardiac pulsation artifacts. Representative images are shown in Figure 4d.

A false-positive rate < 2% was found in the spiral UTE and CS-VIBE sequences (Table 4). Of the false-positive nodules identified by spiral UTE and CS-VIBE sequences, most were close to the diaphragm or around the heart, categorized as small bronchi or vessels by CT images. The spiral UTE and CS-VIBE sequences led to an average false-negative rate of 19% and 17%, respectively, and the missed lesions were considered small in size (average 5.1 mm; range 4–7 mm for spiral UTE, and average 5.3 mm; range 4–8 mm for CS-VIBE images).

### 3.5. Inter-Reader and Inter-Modality Reliability Analysis

**Figure 5:**
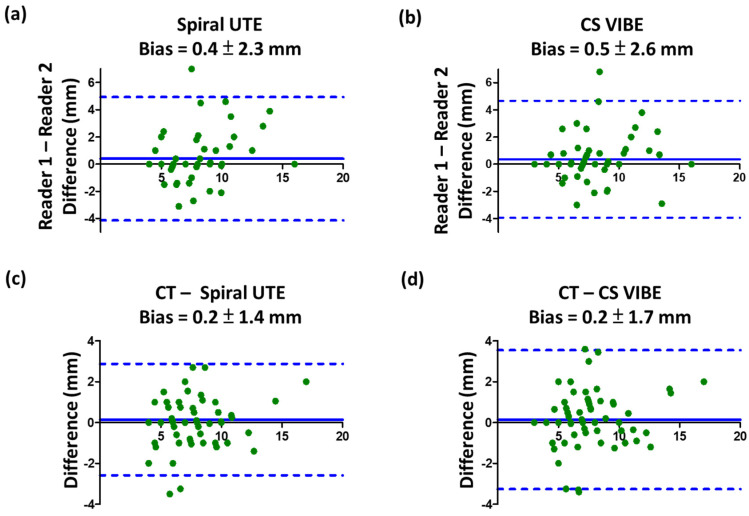
Bland–Altman plots depicting reliability and agreement of CT and MRI nodule measurements. Inter-reader reliability for the nodule diameter measurements showed a small inter-reader bias of less than 0.5 mm for (**a**) spiral UTE and (**b**) CS-VIBE. MRI modalities by (**c**) spiral UTE and (**d**) CS-VIBE images minimally underestimated the nodule size by 0.2 mm, compared to the reference CT. Abbreviations: MR: magnetic resonance; VIBE: volumetric interpolated breath-hold examination; CS: compressed sensing.

In evaluating pulmonary nodule detection, the inter-reader agreement was considered moderate for CS-VIBE, with Cohen’s kappa coefficient κ = 0.537, and substantial for spiral UTE sequence, with κ = 0.672. Inter-reader reliability for nodule diameter measurements showed an inter-reader bias of 0.4 ± 2.3 mm (95% limits of agreement −4.1 to 4.9 mm) for spiral UTE and 0.5 ± 2.6 mm (95% limits of agreement −3.9 to 4.7 mm) for CS-VIBE. The Bland–Altman plots depicting inter-reader agreement are shown in Figure 5a,b.

The inter-sequence reliability for the detection of pulmonary nodules between spiral UTE and CS-VIBE was substantially greater for both readers: κ = 0.639 for reader 1 and κ = 0.661 for reader 2. Nodule size measurements were aligned between CT and MR images. Nodule size measurements from spiral UTE and CS-VIBE images compared with CT images were underestimated by 0.2 ± 1.4 mm (95% limits of agreement −2.6 to 2.9 mm) and 0.2 ± 1.7 mm (95% limits of agreement −3.3 to 3.5 mm), respectively. The Bland–Altman plots depicting inter-modality agreement are shown in Figure 5c,d.

## 4. Discussion

To the best of our knowledge, this is the first study to evaluate the ability of free-breathing spiral UTE and breath-hold CS-VIBE sequences to detect small lung nodules on a 1.5T MRI scanner. Our results demonstrated that both spiral UTE and CS-VIBE images achieved significantly higher image quality scores and lung nodule detection rates than conventional VIBE. The nodule detection rate for spiral UTE and CS-VIBE reached 95% and 100% for nodules >8 and >10 mm, respectively, and 90% for nodules of all sizes with a part-solid or solid morphology. Nodule size measurements were aligned between CT and MR images with minimal deviations of 0.2 mm.

Both UTE and CS-VIBE sequences demonstrated detection ability for small pulmonary nodules. The nodule detection rate in this study was equivalent to that in previous studies reporting detection rates for UTE, zero echo time (ZTE), and radial VIBE MRI. Burris et al. described the nodule detection rate as 83% for UTE [16], whereas Bae et al. demonstrated a nodule detection rate of 89% for ZTE and 84% for UTE [15], and Yu et al. demonstrated a nodule detection rate as 94% for radial VIBE in comparison to 64% for conventional VIBE [17]; missed nodules in the above-mentioned reports were prone to respiratory and cardiac motions, largely mirroring our findings. The high spatial resolution and the shorter TE in CS-VIBE resulted in higher detection rates of lung nodules since a short TE contributes to the detection of non-solid nodules composed of low proton density and a short T2* air–tissue interface. In contrast, the longer TE of conventional VIBE is attributed to its lower detection rate for non-solid nodules.

Importantly, nodule size is more critical than morphology when using MRI to detect pulmonary nodules. In our study, as shown in Figure 2, the nodule detection sensitivity increased proportionately with increasing nodule size; however, it decreased from part-solid to solid nodule groups. The winding trend from part-solid to solid nodules may be due to nodule size variations. The average size of the part-solid nodules was 9.9 mm (range: 4–20 mm), larger than that of solid nodules (mean: 5.7 mm; range, 3–11 mm).

Our study showed that CS-VIBE may bring prominent cardiac motion in a few cases, which may appear from the Cartesian k-space trajectories of CS-VIBE acquisition or ECG gating unequipped. Recently, MR sequences have been combined with multidimensional CS reconstruction approach (eXtra-Dimensional Golden-Angle Radial Sparse Parallel, XD-GRASP) [26,27]. Concatenating the cardiac cycle and respiratory dimension by XD-GRASP for VIBE sequence can be applied to improve cardiac motion artifacts. 

Undeniably, CS-VIBE’s prominent cardiac motion artifact is an impediment to nodule detection in the LLL and RML regions; however, this challenge may be overcome by self-directed learning, experienced interpretation, professional training, and case-based feedback education. In our studies, experienced radiologists with >20 years experience in chest MRI interpretation demonstrated a better diagnostic accuracy and nodule detection rate in the easily missed LLL and RML zones in CS-VIBE and spiral UTE sequences, leading to implications for the design of chest MRI training initiatives, as well as for the possibility of novice radiologists providing clinical chest MR image interpretations. 

Although the average bias was small (< 0.5 mm) in size measurements when considering the inter-reader agreement (reader 1 vs. reader 2) or the inter-sequence reliability (CT vs. spiral UTE or CS-VIBE), the 95% limits of agreement ranged ± 5 mm; this potential measurement differences can change a baseline Lung-Reporting and Data System (Lung-RADS) grade or determine nodule growth between scans.

We previously published a preliminary report on free-breathing spiral UTE sequence for the detection of small lung nodules with real scan times of 3.5–5 min [21]. Spiral UTE provided credible image quality and nodule detection sensitivity; nevertheless, due to the scarcity and uncommon availability of the spiral UTE sequence, the more commonly-available CS-VIBE sequence for lung nodule detection was further incorporated [28]. Our study provided evidence that spiral UTE and CS-VIBE sequences were capable of detecting small lung nodules with acceptable image qualities; however, their clinical application requires further investigation. A comparison table highlighting the advantages and disadvantages of the proposed methods is provided in Table 5.

Besides lung nodule detection, recent advances in MRI techniques, such as UTE and CS-VIBE, have expanded clinical opportunities for pulmonary MRI for cystic fibrosis and pulmonary hypertension [29]. However, their potential in imaging pulmonary embolism, pulmonary parenchymal abnormalities, chronic obstructive pulmonary disease, asthma, or interstitial lung disease is still under investigation, with evident preliminary data [30]. The lack of radiation makes pulmonary MRI an ideal modality for pediatric examinations, pregnant women, and patients requiring serial and longitudinal follow-up. In pediatric patients, the clinical indications involve the evaluation of lung air perfusion patterns in pulmonary hypertension, early detection and follow-up for asthma, cystic fibrosis, pulmonary embolism, and bronchiolitis obliterans [31]. In addition, pulmonary MRI sequences could be included in the body MRI sequence for patients who need thorough evaluation of both the lungs and body organs in a single imaging session.

Our study has several limitations. First, the present series contained observer bias because all lung MRIs were performed for patients scheduled to undergo VATS, and radiologists were aware that only patients with lung nodules would receive MRI scans, which may have inflated the nodule detection sensitivity values. Second, the number of patients and nodules was relatively small; additional studies are needed to determine the diagnostic performances of CS-VIBE and spiral UTE imaging in a larger cohort of patients with varying nodule presentations and characteristics.

There are several notable differences between our study and previous studies in terms of pulmonary nodule detection using MRI. First, the pathology was confirmed in 76% of the nodules. Most (89%) dissected nodules were invasive carcinoma or pre-invasive cancer, necessitating MRI for pulmonary nodule detection in patients not suitable for or unwilling to undergo CT. Second, in our study, respiratory motion managements were compared directly, using free-breathing in the UTE sequence and breath-hold in the CS-VIBE sequence; both demonstrated reliable imaging quality. Therefore, in patients who experience dyspnea and cannot correctly hold their breath or in pediatric patients, using the UTE sequence may reliably decrease the likelihood of respiratory image degradation, whereas in patients who can accurately perform breath-hold, CS-VIBE may be implemented with a short scan time averaging 13 s.

## 5. Conclusions

We have demonstrated the feasibility of both CS-VIBE and spiral UTE in lung imaging and the detection of pulmonary nodules, with an overall nodule detection rate of above 80%, and a 100% detection rate for nodules ≥10 mm, which was clinically meaningful. Both CS-VIBE and spiral UTE MRI could be considered as a potential alternative to chest CT in young people, pregnant women, patients requiring serial and longitudinal follow-up, or people unwilling to undergo radiation exposure.

## Figures and Tables

**Table 1:** Parameters of conventional VIBE, spiral UTE, and CS-VIBE sequences in lung MR images.

Parameters	VIBE	Spiral UTE	CS-VIBE
**TR**	3.90 ms	3.72 ms	4.13 ms
**TE**	1.30 ms	0.05 ms	0.84 ms
**Flip angle**	5°	5°	5°
**Voxel matrix**	1.0 × 1.0 × 3.0 mm^3^	1.56 × 1.56 × 1.56 mm^3^	1.2 × 1.2 × 1.6 mm^3^
**Scan time**	11 s	3.5–5 min, depending on the patient’s breathing pattern	13 s
**Acquired orientation**	Transverse	Coronal	Transverse
**Respiratory trigger**	No	No	No
**Acceleration factor**	CAIPIRINHA iPAT = 3	Spiral iPAT = 2	Acceleration = 5 Iteration = 35

Abbreviations: MR: magnetic resonance; VIBE: volumetric interpolated breath-hold examination; CAIPIRINHA: controlled aliasing in parallel imaging results in higher acceleration; UTE: ultrashort echo time; CS: compressed sensing; TR: repetition time; TE: echo time.

**Table 2:** Patient and nodule characteristics.

**Patient (n = 71)**		
Median age (range) in years		60 (33–81)
Gender (male/female)		(31/40)
**Number of nodules per patient**		
	1	50 (71%)
	2	18 (25%)
	3	2 (3%)
	4	1 (1%)
**Nodule (n = 96)**		
Mean diameter (range), mm		7.7 ± 3.9 (4–20)
**Number of nodules per size category**		
	<6 mm	28 (29%)
	≥6–<8 mm	28 (29%)
	≥8–<10 mm	19 (20%)
	≥10 mm	21 (22%)
**Number of nodules per location**		
	RUL	35 (36%)
	RML	11 (11%)
	RLL	16 (17%)
	LUL	17 (18%)
	LLL	17 (18%)
**Number of nodules per morphology category**		
	Non-solid	43 (45%)
	Part-solid	35 (36%)
	Solid	18 (19%)
**Number of nodules per surgical pathology ^a^ (n = 73)**		
	Invasive adenocarcinoma	27 (37%)
	Minimally invasive adenocarcinoma	26 (36%)
	Squamous cell carcinoma	1 (1%)
	Adenocarcinoma in situ	11 (15%)
	Atypical adenomatous hyperplasia	2 (3%)
	Idiopathic neuroendocrine cell hyperplasia	1 (1%)
	Other benign lesions ^b^	5 (7%)

^a^ Of 96 nodules, 73 were resected by video-associated thoracoscopic resection and examined by a thoracic pathologist. ^b^ Other benign lesions were intrapulmonary lymph nodes, fibrotic bronchitis, cryptococcosis, and hyalinized nodules. Abbreviations: RUL: right upper lobe; RML: right middle lobe; RLL: right lower lobe; LUL: left upper lobe; LLL: left lower lobe.

**Table 3:** Qualitative assessment of normal structures and overall diagnostic acceptability of lung MR images.

Scores (mean ± SD)	VIBE	Spiral UTE	*p*-Value ^a^ (VIBE vs. Spiral UTE)	CS-VIBE	*p*-Value ^a^ (VIBE vs. CS-VIBE)	*p*-Value ^a^ (Spiral UTE vs. CS-VIBE)
Pulmonary vascular depiction	3.3 ± 0.5	3.7 ± 0.6	<0.001	3.8 ± 0.5	<0.001	0.469
Airway depiction	3.0 ± 0.2	3.5 ± 0.6	<0.001	3.3 ± 0.5	<0.001	0.183
Cardiac motion artifact	3.3 ± 0.6	3.8 ± 0.4	<0.001	3.1 ± 0.6	0.056	0.054
Image noise for nodule detection	2.8 ± 0.8	3.7 ± 0.7	<0.001	3.4 ± 0.7	<0.001	0.159
Overall image quality	3.0 ± 0.4	3.7 ± 0.6	<0.001	3.5 ± 0.6	<0.001	0.439

Abbreviations: MR: magnetic resonance; VIBE: volumetric interpolated breath-hold examination; UTE: ultrashort echo time; CS: compressed sensing. ^a^ *p*-values were calculated using Student’s paired-samples *t*-test.

**Table 4:** Assessment of nodule detection capability of lung MR images.

		Sensitivity (%)	Specificity (%)	False Positive Rate (%)	False Negative Rate (%)	Positive Predictive Value (%)	Negative Predictive Value (%)	*p*-Value *
Reader 1	VIBE	50/96 (52.1)	251/259 (96.9)	8/259 (3.1)	46/96 (47.9)	50/58 (86.2)	251/297 (84.5)	-
	Spiral UTE	79/96 (82.3)	256/259 (98.8)	3/259 (1.2)	17/96 (17.7)	79/82 (96.3)	256/273 (93.8)	<0.001 ^a^
	CS-VIBE	82/96 (85.4)	257/259 (99.2)	2/259 (0.8)	14/96 (14.6)	82/84 (97.6)	257/271 (94.8)	0.004 ^a^ 0.629 ^b^
Reader 2	VIBE	51/96 (53.1)	252/259 (97.3)	7/259 (2.7)	45/96 (46.9)	51/58 (87.9)	252/297 (84.8)	-
	Spiral UTE	76/96 (79.2)	257/259 (99.2)	2/259 (0.8)	20/96 (20.8)	76/78 (97.4)	257/277 (92.8)	<0.001 ^a^
	CS-VIBE	77/96 (80.2)	258/259 (99.6)	1/259 (0.4)	19/96 (19.8)	77/78 (98.7)	258/277 (93.1)	<0.001 ^a^ 1.000 ^b^

Abbreviations: MR: magnetic resonance; VIBE: volumetric interpolated breath-hold examination; UTE: ultrashort echo time; CS: compressed sensing. * The *p*-value was calculated using McNemar’s test to evaluate the nodule detection rate compared to ^a^ conventional VIBE or ^b^ spiral UTE.

**Table 5:** Advantages and disadvantages of the proposed imaging methods.

	Advantages	Disadvantages
Chest CT	Gold standard for lung nodule detection; nodule detection sensitivity 100%. Gold standard for normal structure depiction; very good image resolution. Short scanning durations of <10 s.	Radiation exposure of 2–7 mSv. Breath-hold required; not suitable for patients who cannot hold their breath.
MRI VIBE	Short scanning durations of 11 s. No radiation exposure; suitable for young people, pregnant women, patients requiring serial and longitudinal follow-up, or people unwilling to undergo radiation exposure.	Low nodule detection sensitivity (53%). Relatively low image quality for normal structures; Relatively low SNR and CNR. Breath-hold required; not suitable for patients who cannot hold their breath.
MRI Spiral UTE	Acceptable nodule detection sensitivity (81%). Good image quality for normal structures; good SNR and CNR. No radiation exposure; suitable for young people, pregnant women, patients requiring serial and longitudinal follow-up, or people unwilling to undergo radiation exposure. Free-breathing acceptable; suitable for patients who cannot hold their breath or pediatric patients.	Relatively long scanning durations of 3.5–5 min.
MRI CS-VIBE	Acceptable nodule detection sensitivity (83%). Good image quality for normal structures; good SNR and CNR. No radiation exposure; suitable for young people, pregnant women, patients requiring serial and longitudinal follow-up, or people unwilling to undergo radiation exposure. Short scanning durations of 13 s.	Breath-hold required; not suitable for patients who cannot hold their breath.

Abbreviations: CT: computed tomography; MRI: magnetic resonance imaging; VIBE: volumetric interpolated breath-hold examination; UTE: ultrashort echo time; CS: compressed sensing; SNR: signal-to-noise ratio; CNR: contrast-to-noise ratio.

## Data Availability

All data analyzed during this study are included in this published article (and its supplementary information files).

## Supplementary Materials

The following supporting information can be downloaded at: https://www.mdpi.com/article/10.3390/diagnostics12010093/s1, Table S1: Qualitative grading system for assessment of lung MR images obtained using conventional VIBE, spiral UTE, and CS VIBE sequences.
