# Supplementary material for: Applying Compressed Sensing Volumetric Interpolated Breath-Hold Examination and Spiral Ultrashort Echo Time Sequences for Lung Nodule Detection in MRI
Source: Diagnostics (Basel). 2021 Dec 31;12(1):93. doi: 10.3390/diagnostics12010093 (PMC8774698; doi:10.3390/diagnostics12010093)
Supplement: Supplementary file 1 [file diagnostics-12-00093-s001.zip › diagnostics-1467027-supplementary.pdf]

Table S1. Qualitative grading system for assessment of lung MR images obtained using conventional VIBE, spiral UTE, and CS VIBE sequences

Score	1	2	3	4	5
Pulmonary vascular depiction	Unacceptable (invisible peripheral pulmonary vessels)	Poor (barely visible peripheral pulmonar y vessels)	Fair (visible peripheral pulmonary vessels)	Good (visible peripheral pulmonary vessels with clear margin)	Excellent (visible peripheral pulmonary vessels to the distal lung periphery)
Airway depiction	Unacceptable (indistinguisha ble lobar bronchial walls)	Poor (visible lobar bronchial walls with few visible segmental bronchial walls)	Fair (visible segmental bronchial walls)	Good (visible subsegmenta l bronchial walls)	Excellent (visible sub-subsegmenta l bronchial walls)
Cardiac motion artifact	Unacceptable artifacts	Moderate artifacts	Acceptable artifacts	Slight artifacts	No artifact
Image noise for nodule detection	Unacceptable noise affecting nodule detection	Moderate noise	Acceptable noise	Slight noise	Minimal or no noise
Overall image quality	Non-diagnostic	Poor	Acceptable	Good	Excellent

Abbreviations: MR: magnetic resonance; VIBE: volumetric interpolated breath-hold examination; UTE: ultrashort echo time; CS: compressed sensing.
